# Identification of sequence motifs significantly associated with antisense activity

**DOI:** 10.1186/1471-2105-8-184

**Published:** 2007-06-07

**Authors:** Kyle A McQuisten, Andrew S Peek

**Affiliations:** 1 Department of Bioinformatics, Integrated DNA Technologies, 1710 Commercial Park Road, Coralville, IA 52241, USA

## Abstract

**Background:**

Predicting the suppression activity of antisense oligonucleotide sequences is the main goal of the rational design of nucleic acids. To create an effective predictive model, it is important to know what properties of an oligonucleotide sequence associate significantly with antisense activity. Also, for the model to be efficient we must know what properties do not associate significantly and can be omitted from the model. This paper will discuss the results of a randomization procedure to find motifs that associate significantly with either high or low antisense suppression activity, analysis of their properties, as well as the results of support vector machine modelling using these significant motifs as features.

**Results:**

We discovered 155 motifs that associate significantly with high antisense suppression activity and 202 motifs that associate significantly with low suppression activity. The motifs range in length from 2 to 5 bases, contain several motifs that have been previously discovered as associating highly with antisense activity, and have thermodynamic properties consistent with previous work associating thermodynamic properties of sequences with their antisense activity. Statistical analysis revealed no correlation between a motif's position within an antisense sequence and that sequences antisense activity. Also, many significant motifs existed as subwords of other significant motifs. Support vector regression experiments indicated that the feature set of significant motifs increased correlation compared to all possible motifs as well as several subsets of the significant motifs.

**Conclusion:**

The thermodynamic properties of the significantly associated motifs support existing data correlating the thermodynamic properties of the antisense oligonucleotide with antisense efficiency, reinforcing our hypothesis that antisense suppression is strongly associated with probe/target thermodynamics, as there are no enzymatic mediators to speed the process along like the RNA Induced Silencing Complex (RISC) in RNAi. The independence of motif position and antisense activity also allows us to bypass consideration of this feature in the modelling process, promoting model efficiency and reducing the chance of overfitting when predicting antisense activity. The increase in SVR correlation with significant features compared to nearest-neighbour features indicates that thermodynamics alone is likely not the only factor in determining antisense efficiency.

## Background

When an antisense DNA oligonucleotide forms a heteroduplex with an mRNA molecule, it triggers a response from RNaseH, which cleaves the heteroduplex [1,2]. The sequence-specificity of this posttranscriptional process makes it theoretically possible to inhibit the expression of any given target gene without inadvertently inhibiting other nearby genes. Such a tool is invaluable for genetic researchers trying to understand the complicated regulatory mechanisms that govern genetic processes. Studies of possible target sites for antisense suppression have shown that not all oligonucleotide sequences are effective in inducing suppression, with many sequences not inducing suppression at all. This kind of exhaustive experimental screening is prohibitively expensive and time consuming, which makes a computational model that relates oligonucleotide sequence to suppression activity desirable. Many computational experiments have been performed to help develop such models [3-14]. When developing these models, it is critical to know which features to include so the model has enough power, but also to know which features not to include because they don't contribute to the classification power of the model. Past studies have tried to associate properties of the antisense oligonucleotide sequence with suppression activity, including the base sequence itself. A small number of subsequence motifs have been identified as associating significantly with antisense activity [7,13], but a broad survey over motifs of different lengths using a publicly available dataset has not been performed until now. For this task, we have used a randomization procedure to assess the association of a large number of motifs with antisense activity.

Previous studies have used several different computational methods to determine which features to include in a model, including *t*-test correlation analysis, mutual information content, and SVM-based recursive feature elimination [13,15]. However, none of these studies implemented a randomization procedure such as the one described in this paper. Also unlike their experiments, this paper will be using publicly available datasets instead of private experimental results supplied by industry. Antisense activity has been correlated to several different properties, including thermodynamic properties of the antisense oligonucleotide sequence [6,16], oligonucleotide secondary structure, structure and accessibility of the mRNA target region [9,12,14,17-19], and motif sequences in the oligonucleotide [13]. Only a small number of such motifs have been studied, and the intent of this study is to survey a wide range of motifs to determine their association with antisense activity. We hypothesize that the thermodynamic properties of the motifs significantly associated with effective antisense suppression will be similar to those previously discovered as effective [6,7,13], with a similar hypothesis for motifs associated with ineffective antisense suppression. That is, we would expect the motifs associated with effective antisense activity to have lower Gibbs free energy (*dG*) than those associated with ineffective antisense activity. Also, while several studies have looked into the association between motif presence and sequence activity, we will attempt to deepen this by analysing the possibility of association between the location of a motif within an antisense sequence and that sequence's activity.

## Results

**Figure 1:**
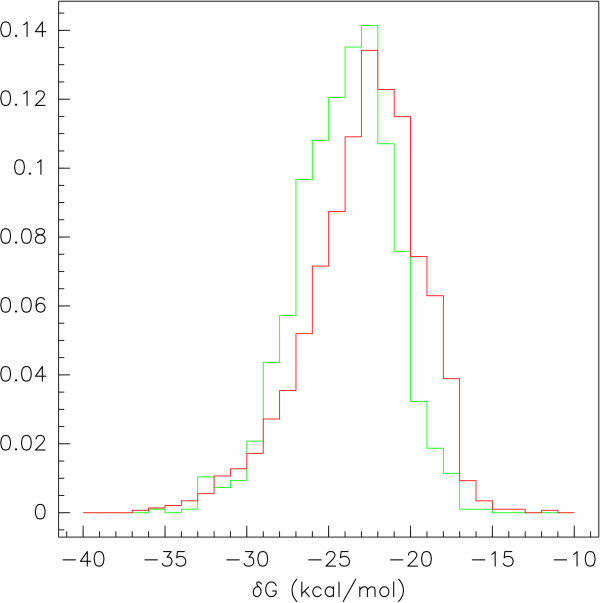
**Thermodynamic Distribution of Experimental Sequences**. Empirical thermodynamic distributions of experimental antisense sequences. The curves represent the distribution of Gibbs free energy (*dG*) for the effective (green) and ineffective (red) antisense sequences. The average *dG *for the effective sequences (-24.04 kcal/mol) was significantly more negative than the average *dG *for the ineffective sequences (-22.80 kcal/mol).

The activities of the antisense sequences ranged from 0.0 to 1.0, where an activity of 0.0 indicates complete inhibition of the target gene and 1.0 is no difference in target activity when compared to the appropriate control. As expected, the distribution of activities is skewed (g_1 _= -0.520, H_0_: g_1 _= 0, t_s _= 13.3, p < 1 × 10^-10^), with fewer sequences having activities near 0.0, as well as highly platykurtic (g_2 _= -0.936), with fewer sequence activities near the mean than when compared to a normal distribution. Even if we remove the 1130 sequences that had an activity of 1.0, the remaining 2783 sequence activities were skewed (g_1 _= -0.233, H_0_: g_1 _= 0, t_s _= 5.02, p = 5e-7), as well as platykurtic, (g_2 _= -0.873). The base composition of the sequences is nonuniform, containing slightly more instances of G and slightly fewer instances of A (A: 22.5%, G: 26.8%, C: 25.7%, T: 25.0%). Thermodynamically, the Gibbs free energy (*dG*) of the effective sequences (μ = -24.04 kcal/mol, σ = 2.94 kcal/mol) was significantly less (*t*-test, *t *= -10.18, p = 4.90e-24) than that of the ineffective antisense sequences (μ = -22.8 kcal/mol, σ = 3.42 kcal/mol). The empirical densities of these thermodynamic distributions are given in Figure 1.

**Figure 2:**
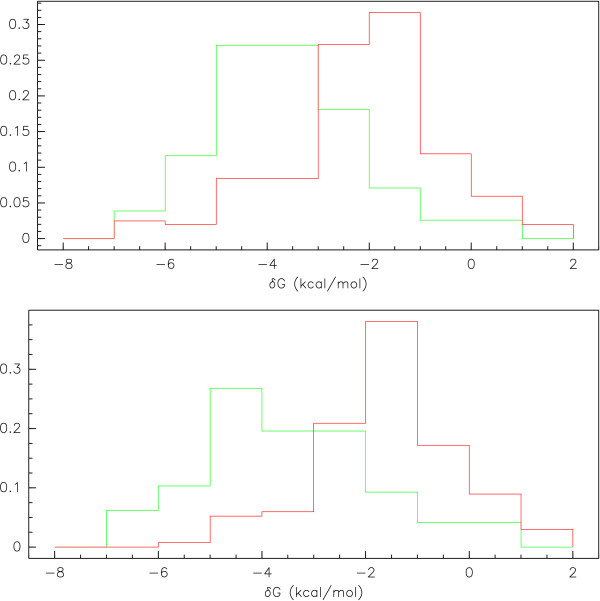
**Thermodynamic Distributions of (top) Significant Motifs and (bottom) Submotif-unique Significant Motifs**. The distributions of Gibbs free energy values (*dG*) for sequences associated with effective antisense activity (green) and those associate with ineffective antisense activity (red). The difference in average *dG *between "good" and "bad" motifs in the subword-unique motifs (-3.45 vs. -1.66 kcal/mol) is greater than the difference in means in the entire population of significant motifs (-3.59 vs. -2.105 kcal/mol). This can be attributed to the removal of motifs from each population that contain submotifs in the opposing group.

After applying our randomization method to the dataset, we found 155 motifs that associated significantly with effective antisense suppression and 202 motifs that were significantly associated with antisense activity, which are presented in Tables 1 and 2. One of the most striking differences between these two sets of motifs is in their base compositions. Motifs that associate with effective antisense suppression are composed of nearly half G, with the remaining percentage split relatively evenly between the remaining bases (G: 48.9%, A: 15.1%, C: 17.3%, T: 18.7%). Those motifs significantly associated with poor antisense activity are quite different, composed of almost no G, but rather dominated by A's and C's (G: 9.5%, A: 39.2%, C: 31.0%, T: 20.3%). This difference in base composition contributes to a marked difference in the thermodynamic properties of these sequences. The average value of *dG *was significantly more negative (*t*-test, *t *= -9.369, p = 1.090e-18) for those motifs associated with effective antisense (μ = -3.593 kcal/mol, σ = 1.465 kcal/mol) than for those associated with poor activity (μ = -2.105 kcal/mol, σ = 1.517 kcal/mol). We also find that the average *dG *of "good" motifs is significantly more negative (*t*-test, *t *= -3.460, p = 6.682e-4) and the average *dG *of the "bad" motifs significantly less negative (*t*-test, *t *= 9.374, p = 4.047e-18) than the average *dG *of the entire population of possible sequence motifs (μ = -3.166 kcal/mol, σ = 1.387 kcal/mol). The thermodynamic distributions of the "good" and the "bad" motifs are given in the top graph of Figure 2.

**Table 1:** Complete list of motifs associated with effective antisense suppression

Motifs associated with effective antisense activity, sorted by length								
2	3	4		5				

CA	ACC	AACC	CCTC	AAACG	CATCG	CCGGG	CTCCC	GTCAC
CC	CAC	AACG	CGCC	AACCA	CATGT	CCGTG	CTCCG	GTCCC
CG	CCA	ACCA	CGCT	AACCC	CCAAC	CCTAC	CTCGT	GTGTC
CT	CCC	ACCC	CGTC	AACGA	CCACC	CCTCC	CTCTC	GTGTG
GC	CCG	ACCT	CGTG	ACCAT	CCACG	CCTGT	CTGTG	TACCA
TC	CCT	ACTC	CTCC	ACCCT	CCACT	CCTTG	GAACG	TACCC
	CGC	AGCC	GCCA	ACCGG	CCATC	CGACC	GACCG	TCCAC
	CGT	AGCT	GCCC	ACCTA	CCCAA	CGACG	GATAG	TCCCC
	CTC	ATCG	GCGT	ACGAA	CCCAC	CGATG	GCAAG	TCCCG
	GCC	CACC	GTCC	ACGCA	CCCAT	CGCAA	GCCAC	TCCCT
	GCT	CACT	TACC	ACGCG	CCCCA	CGCCC	GCCTC	TCCGC
	TCC	CATC	TCCC	ATACC	CCCCC	CGCCT	GCGAC	TCCGT
	TGC	CCAC	TCCG	ATCCC	CCCGC	CGCGA	GCGCT	TCGTC
		CCAT	TCGT	ATCGC	CCCTC	CGGTA	GCGTC	TCTCG
		CCCA	TCTC	ATCTC	CCCTG	CGTAC	GCGTT	TGATA
		CCCC	TGTC	CAACC	CCCTT	CGTCC	GCTAA	TGCGC
		CCCG	TGTG	CAAGC	CCGAC	CGTGA	GGCCA	TGTGT
		CCCT	TTGC	CACCA	CCGCC	CGTGC	GGCGT	TTGCG
		CCGC		CACTC	CCGCG	CTACC	GGGCC	TTGGC
				CATCC	CCGCT	CTCAG	GGTCC	

**Table 2:** Complete list of motifs associated with ineffective antisense suppression

Motifs associated with ineffective antisense activity, sorted by length								
2	3	4		5				

AA	AAA	AAAA	GCGG	AAAAA	AGAAA	CGATA	GGGGC	TATGA
AT	AAG	AAAG	GGAA	AAAAG	AGGGA	CGCTA	GGGGG	TATTA
GA	AAT	AAAT	GGAG	AAACA	AGGGG	CGGGG	GGGGT	TATTG
GG	AGG	AACA	GGGA	AAAGA	ATAAA	CGGTT	GGTAG	TATTT
TA	ATA	AAGA	GGGG	AAAGT	ATAAC	CGTAA	GGTTA	TCAAA
TT	ATT	AATA	GTTA	AAATA	ATAAT	CGTGT	GTAAG	TCAAT
	GAA	AATC	TAAA	AAATG	ATACG	CTATA	GTAGG	TCTAG
	GGA	AATG	TAAT	AAATT	ATAGA	CTATC	GTGGG	TCTTA
	GGG	AATT	TACG	AACAA	ATAGG	CTCAC	GTGTA	TGGAT
	TAA	ACAA	TACT	AACTA	ATATA	CTGGG	GTTAT	TGGGG
	TAG	ACTA	TAGA	AAGAA	ATATT	CTTAC	TAAAA	TTAAA
	TAT	AGAA	TAGG	AAGAT	ATCCG	CTTCG	TAAAC	TTAAC
	TTA	AGAT	TATA	AAGCG	ATCGA	GAAAA	TAAAG	TTAAT
	TTT	AGGA	TATT	AATAA	ATTAA	GAAAG	TAAAT	TTACA
		AGGG	TCAA	AATAG	ATTAG	GAATA	TAATA	TTACG
		ATAA	TCTA	AATAT	ATTAT	GACGA	TAATC	TTACT
		ATAT	TCTT	AATCA	ATTGA	GATAA	TAATG	TTAGT
		ATTA	TGGG	AATCT	ATTTA	GATTA	TAATT	TTATA
		ATTT	TTAA	AATGG	ATTTG	GCGCG	TACGT	TTATT
		CAAT	TTAC	AATTA	ATTTT	GCGGC	TACTT	TTCGA
		CATA	TTAT	AATTG	CAAAT	GCGGG	TAGAA	TTCTT
		GAAA	TTCG	AATTT	CAATA	GGAAT	TAGAT	TTTAA
		GACG	TTTA	ACAAA	CATTA	GGACA	TAGGT	TTTAC
		GATT	TTTT	ACAAT	CATTT	GGATC	TAGTT	TTTAT
				ACATA	CCGGC	GGGAC	TATAA	TTTTA
				ACTAC	CGAGC	GGGCG	TATAT	TTTTT
				ACTAT	CGAGT	GGGGA	TATCA	

When examining the relationship between motif position and antisense effectiveness, we note that of the 167 contingency tables calculated for motifs associated with antisense effectiveness, 130 of them had sufficiently large entries for the chi-square test. Of these 130, only two (1.53%) had large enough values of the chi-square statistic to reject the null hypothesis of independence between the axes of the table (GCGCT: X = 8.15, p = 4.3e-3; CGTC: X = 9.64, p = 1.9e-3). Similarly, of the 202 motifs associated with ineffective antisense activity, 63 had large enough table counts, only one (1.59%) of which had a significant value for the t-statistic (TGGG: X = 5.84, p = 0.015). When the Bonferronni correction for multiple comparisons is applied to these p-values, none of them remain significant.

**Figure 3:**
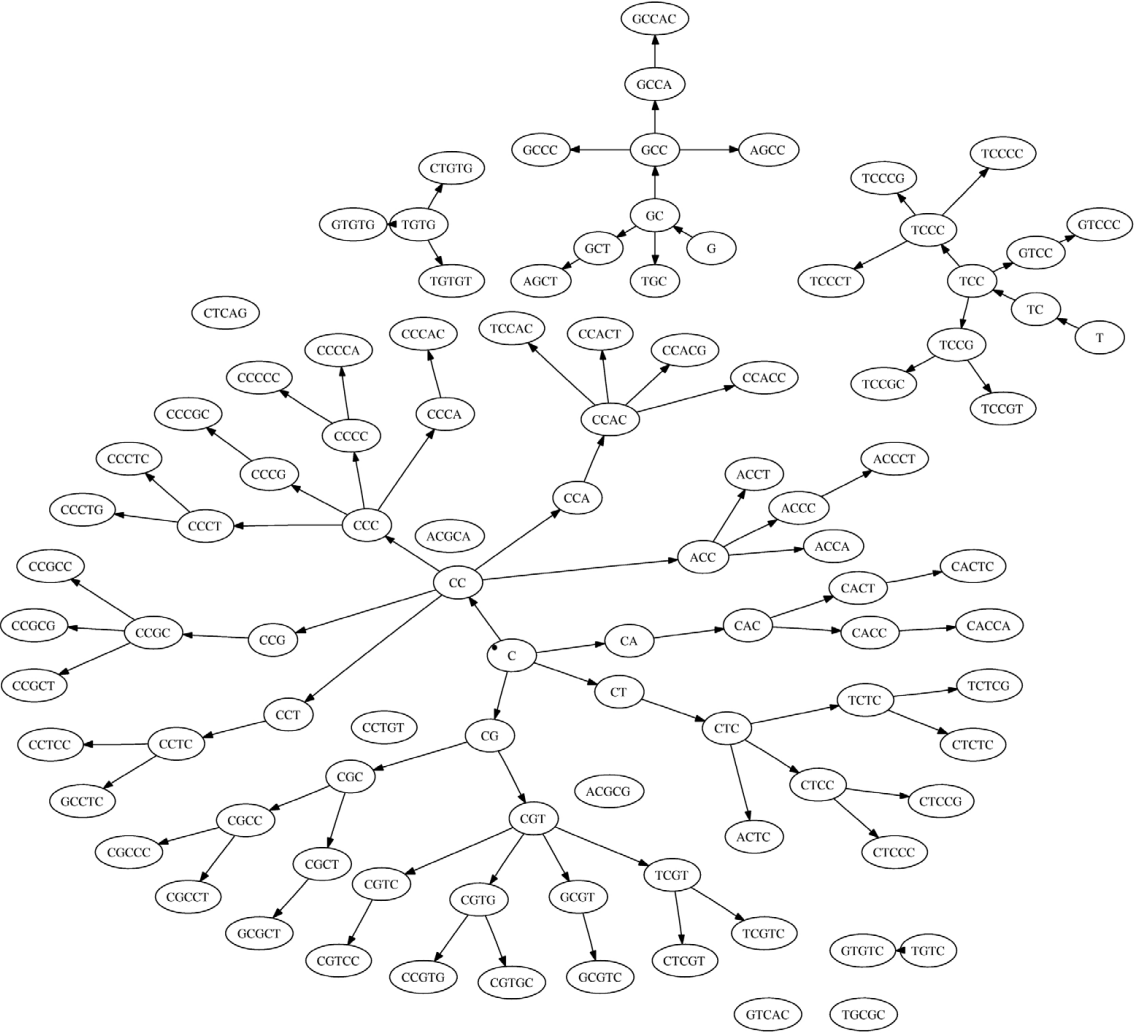
**Subword Structure of Effective Subword-unique Motifs**. A subword tree illustrating the makeup of the subword-unique motifs associated with antisense effectiveness. A motif is linked with an arrow to another if the motif at the tail of the arrow is a submotif of the one at the head that differs only by the addition of one base to the beginning or the end. For example, "CC" would be at the tail of an arrow connecting it to either "CCG" or "GCC", but there would be no arrow connecting it to "CGC" or "GGCC". For motifs associate with antisense effectiveness, the majority of significant motifs are linked in a tree with "C" as the root node.

**Figure 4:**
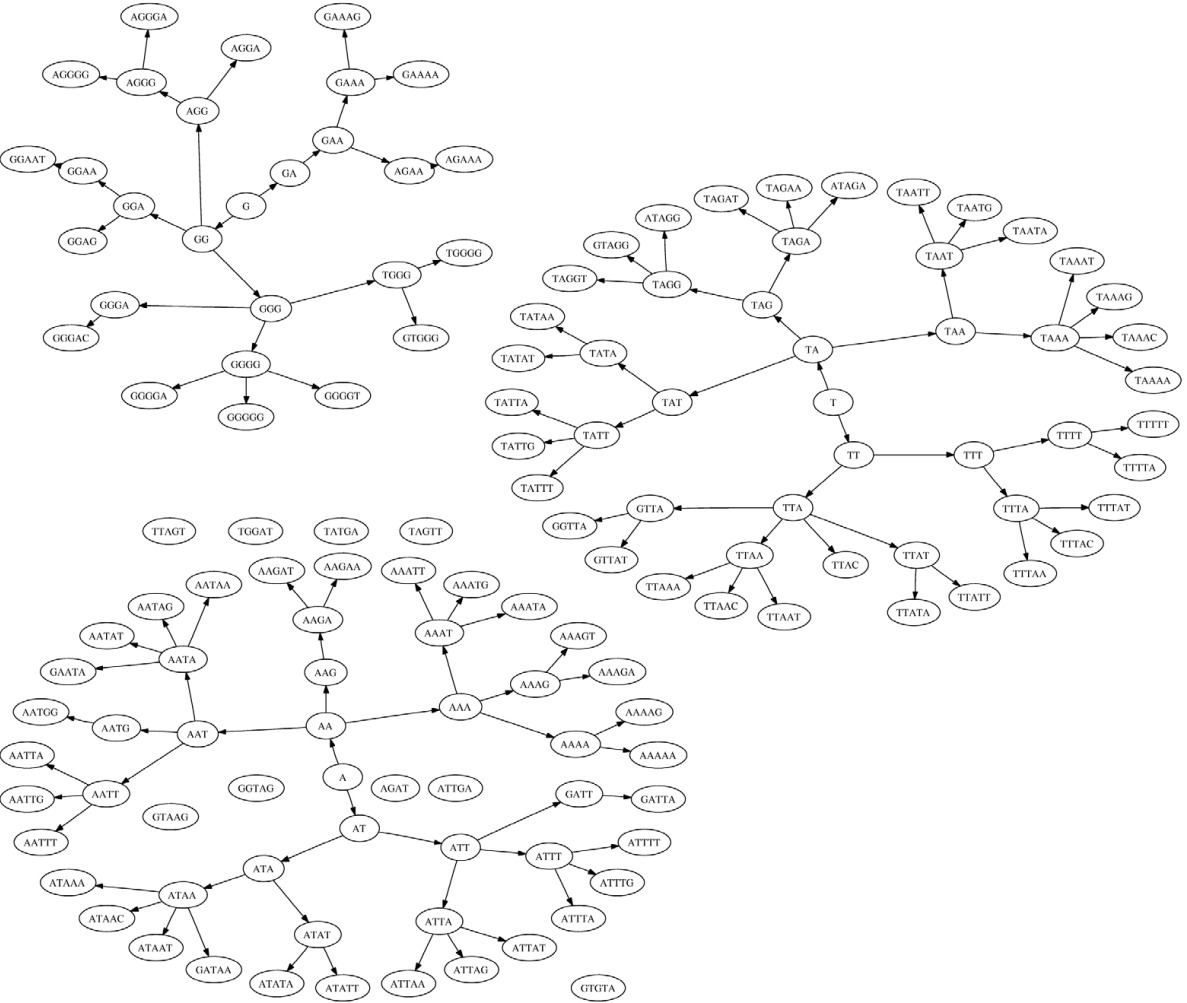
**Subword Structure of Ineffective Subword-unique Motifs**. A subword tree constructed in the same manner as Figure 3, but for motifs associated with ineffective antisense suppression. In this population of motifs, nodes are grouped into two main trees, one rooted with "A", one rooted with "T". Surprisingly, despite the lower Gibbs free energy associated with its presence, a third tree rooted at "G" was found within this population of motifs as well.

Another feature of the set of significant motifs found by our method was the subword structure of the motifs found. Many of the significant motifs discovered were contained in longer motifs that were also found to be significant. This is illustrated by the directed graphs given in Figure 3 and Figure 4. When looking at the subword makeup of the significant motifs, we found that of the 167 motifs associated with effective antisense activity, 70 of them contained submotifs that were associated with ineffective antisense activity. Conversely, of the 202 motifs associated with ineffective antisense activity, 68 contained submotifs that were significantly associated with effective antisense activity. When looking at the remaining population of subword-unique motifs associated with effective and ineffective antisense activity, we see the expected differences in thermodynamic properties. Thermodynamic distributions of the subword-unique motifs are shown in the bottom graph of Figure 2. The subword-unique motifs associated with antisense effectiveness (μ = -3.45 kcal/mol, σ = 1.66 kcal/mol) have a significantly more negative *dG *(*t*-test, *t *= -9.07, p = 2.32e-16) than those associated with antisense ineffectiveness (μ = -1.66 kcal/mol, σ = 1.301 kcal/mol). Furthermore, while there was no significant thermodynamic differences between the subword-unique good motifs and the entire population of good motifs, the subword-unique motifs associated with antisense ineffectiveness had significantly larger values of *dG *compared to the entire population of motifs associated with antisense ineffectiveness (*t *= 3.20, p = 0.0015). This can likely be attributed to the removal of motifs containing 'GC' and 'CG' as submotifs, thus making the average *dG *values for the motifs less negative.

Support vector regression tests were carried out in two forms: training and testing on the entire dataset and 10-fold cross validation, with correlation and MSE results presented in Table 3. When the SVR was trained on the entire dataset and used to predict the entire dataset, the correlation induced by the feature set of all possible motifs significantly outperformed all other feature sets. However, when 10-fold cross validation was applied, the set of significant motifs outperformed the other feature sets, including the set of all possible motifs. The values of the *t*-statistic and the associated *p*-values for the pairwise comparisons of correlation coefficients are given in Table 4 and Table 5, respectively.

**Table 3:** Support Vector Regression Results for Motif Significance

SVR Test Results, N = 3868		
Train All		

	MSE	r

All	0.061	0.432
Sig	0.065	0.364
gSig	0.069	0.282
bSig	0.069	0.282
uSig	0.066	0.339
guSig	0.070	0.264
buSig	0.071	0.243

10-fold CV		

	MSE	r

All	0.069	0.289
Sig	0.068	0.307
gSig	0.072	0.228
bSig	0.072	0.217
uSig	0.069	0.286
guSig	0.072	0.224
buSig	0.072	0.196

**Table 4:** *t*-statistic values for correlation coefficient significance between feature sets

*t*-statistic values for correlation coefficient significance						
Entire Dataset						

	All	Sig	gSig	bSig	uSig	guSig
All						
Sig	8.743					
gSig	14.168	5.425				
bSig	14.377	5.634	0.208			
uSig	12.420	3.677	-1.748	-1.956		
guSig	16.111	7.368	1.943	1.735	3.691	
buSig	17.125	8.382	2.957	2.748	4.705	1.014

10-fold Cross Validation						

	All	Sig	gSig	bSig	uSig	guSig
All						
Sig	-2.176					
gSig	1.184	3.360				
bSig	1.103	3.279	-0.081			
uSig	0.343	2.529	-0.841	-0.760		
guSig	2.183	4.359	0.999	1.081	1.841	
buSig	3.556	5.732	2.372	2.454	3.214	1.373

**Table 5:** *p*-values for correlation coefficient significance between feature sets

*p*-values for correlation coefficient significance						
Entire Dataset						

	All	Sig	gSig	bSig	uSig	guSig
All						
Sig	1					
gSig	1	1				
bSig	1	1	0.583			
uSig	1	1	0.040	0.025		
guSig	1	1	0.973	0.958	1	
buSig	1	1	1	1	1	0.844

10-fold Cross Validation						

	All	Sig	gSig	bSig	uSig	guSig
All						
Sig	0.015					
gSig	0.881	1				
bSig	0.864	1	0.468			
uSig	0.633	0.994	0.200	0.224		
guSig	0.985	1	0.841	0.140	0.967	
buSig	1	1	0.991	0.993	1	0.915

An additional set of SVR models were constructed to test the hypothesis that antisense is a primarily thermodynamically driven process. The previously mentioned thermodynamic calculations were made using nearest-neighbour thermodynamic parameters. These parameters correspond to motifs of length two, so we hypothesized that if these thermodynamic values were the driving factor in antisense efficacy, then an SVR model using only these length two motifs would have strong predictive power, and the inclusion of longer motifs would reduce predictive power and increase overfitting. To test this, we trained 10-fold cross-validated SVR models on the set of all motifs, the set of all 2-mers, the set of significant motifs, and the set of significant 2-mers. The values for the correlation coefficient and the MSE of these predictions are given in Table 6, and *t*-statistics and p-values for the comparison of those correlations are presented in Table 7.

**Table 6:** Support Vector Regression Results for Significance vs. NN-thermodynamics

SVR Test Results, N = 3868		
10-fold Cross-validation		

	MSE	r

Sig	0.067	0.330
NN	0.070	0.270
NNSig	0.070	0.269

**Table 7:** *t*-statistics and *p*-values for correlations between significant feature sets and nearest-neighbour feature sets

*t*-statistic and *p*-values for correlation significance		
*t*-statistics		

	Sig	NN

Sig		
NN	2.917	
NNSig	2.969	0.052

*p*-values		

	Sig	NN

Sig		
NN	0.998	
NNSig	0.998	0.521

## Discussion

In order to complete our desired task of determining motifs that significantly associate with antisense effectiveness/ineffectiveness, we developed a Monte Carlo randomization procedure to systematically determine if a wide range of motifs were overrepresented in either effective or ineffective antisense sequences. Using this method, we were able to confirm the association of several previously discovered motifs with antisense effectiveness, such as TCCC [7,13], CCAC, ACTC, and GCCA. However, the motif CTCT, which had been previously identifies as associating significantly with antisense effectiveness, was not determined to be significant by our method. In addition, our method confirmed the association of sequence motifs such as GGGG, TAA, and AAA with antisense ineffectiveness [7]. The motif ACTG, which had been found to associate significantly with antisense ineffectiveness [7], was not found to significantly associate by our method. In addition to these previously discovered significant motifs, we uncovered hundreds of motifs that appear to associate significantly with antisense activity.

This seemingly large number of new motifs could be the result of the "wide net" cast to determine which sequences were considered effective and ineffective. By classifying all sequences with activity below 0.5 as effective and all those above as ineffective, we are conflating both very effective and only moderately effective sequences. Similarly, we are conflating the totally ineffective sequences with those that do show some noticeable knockdown activity. This may lead to common motifs in the moderately active (activity 0.25 to 0.75) sequences being associated significantly with either positive or negative antisense activity. We predict that a modification of the randomization method classifying only very active (activity < 0.25) and very inactive (activity > 0.75) sequences will result in a set of significant motifs that is a subset of the one presented here, and that that those motifs that are overrepresented in only the moderately effective antisense sequences (0.25 < activity < 0.75) would not appear in this refined set of significant motifs. We theorize that once these statistical/mathematical artefacts are accounted for, the resulting sets of significant motifs will have even greater thermodynamic differences than those presented here, reinforcing the hypothesis that antisense suppression is heavily driven by probe/target thermodynamics.

Also, due to the correlation between local target secondary structure and suppression efficacy, we are concerned that the association of motifs with effective antisense suppression is driven by bias in local target structure. To determine if this were the case, we could perform our randomization method on the regions of our targets where secondary structure promotes or inhibits binding. If target secondary structure were driving sequence activity, we would expect to find the complements of our significant motifs overrepresented in these regions.

It appears that the population of motifs that associate significantly with antisense activity are not chosen at random, as there are similarities in both thermodynamic properties and subword-structures. This is to be expected since biological systems are certainly not constructed randomly, but it does seem to indicate that these statistical methods can identify physical properties that may be important factors in antisense effectiveness. It is worth noting, however, that while statistical significance may help us to identify physical properties, it may not directly imply biological relevance. We have found significant association between antisense activity and the thermodynamic properties of the motifs composing an antisense sequence, which is consistent with previous studies associating sequence thermodynamics and antisense activities. However, the thermodynamic analysis of the motifs identified by our method may lend us more information about the nature of the link between activity and thermodynamics. The difference in *dG *between the ineffective motifs and the overall motif population is approximately 2.2 times the difference between the *dG *of effective motif sequences and the overall population of possible motifs. This may indicate that the key factor in thermodynamic design of antisense sequences may lie more in the avoidance of motifs with poor thermodynamic characteristics rather than intentionally choosing motifs with favourable characteristics.

Surprisingly, we have also found that the positions of effective motifs within an antisense sequence do not have any significant statistical association with antisense activity. This may also serve to indicate that the specific motifs may not be as important to antisense activity as having thermodynamically helpful motifs uniformly distributed across an antisense sequence.

One limitation of our Monte-Carlo method is that it does not address the nature of the subword structure of the motifs found to associate with antisense activity. Any time a motif is found and counted in our method, any submotif of that motif will by definition be counted as well. However, we showed that there were instances of effective motifs containing submotifs that associate with ineffective sequences. When a motif is determined to be overrepresented in the empirical dataset for a particular iteration, it is not necessarily the case that a given submotif will also be overrepresented, as the random dataset could have many instances of the submotif while having few instances of the original motif. Future work using our method may include modifications to include calculations that will determine if there is any connection between the significance of submotifs and the significance of motifs comprised of these submotifs.

In the SVR regression tests, the large drop in correlation under cross-validation for the "All Motifs" feature set indicates that the high performance seen initially is likely the result of overfitting, whereas the relatively consistent performance of the set of "Significant Motifs" leads us to believe it is a better feature set to use for modelling the activity of unknown antisense oligos. Also of note is the drop in effectiveness for the split feature sets "gSig", "bSig", "guSig", and "buSig". This seems to indicate that basing a model on motifs associated exclusively either with positive or negative activity is not sufficient for modelling the entire population of both effective and ineffective antisense sequences, and that both types of significant motifs should be used in the model.

When comparing the performance of SVR regression using only 2-mers as features to those using significant motifs of length 2 to 5, we find that the feature set of significant motifs vastly outperforms the set of 2-mers (*t*-test, p = 0.998) as well as the set of significant 2-mers (*t*-test, p = 0.998), while the set of 2-mers and significant 2-mers were not statistically different from one another (*t-*test, p = 0.521). This leads us to conclude that while thermodynamics is certainly driven by nearest-neighbour thermodynamics, the increased predictive power of longer motifs in the SVR model suggests that there are biological mechanisms other than thermodynamics contributing to the efficacy of sequences. We suspect that this may be related to the size of the region of the DNA-RNA duplex that RNaseH interacts with while bound to the duplex.

## Conclusion

This paper describes a Monte-Carlo method for analysing the association of a broad range of sequence motifs to antisense suppression activity. We have discovered a collection of previously unstudied sequence motifs that associate significantly with antisense activity, thus giving researchers a larger pool of features from which to base motif-driven sequence-activity models for antisense suppression. This allows flexibility in effective antisense design, and gives researchers more opportunity to design oligonucleotides containing motifs that were previously not known to increase antisense activity and use targets that were not known to be effective binding regions for antisense suppression. Our results confirm previous notions of the relationship between oligonucleotide thermodynamics and antisense suppression efficiency, but also extend them, as thermodynamics alone are unable to explain the increased predictive power of longer motifs. We have also illustrated the independence of motif position with antisense activity, allowing for simpler sequence-activity models that can run more efficiently and risk less overfitting due to feature redundancy.

When we apply our results to an SVR model, we see examples of the overfitting displayed by models using too many features as well as significant improvements in prediction correlation when using our set of significant motifs is used as the base for the SVR model. Since we used a cut-off value to classify our original data, we hope to improve results for SVR by using different cut-off values for high and low activity sequences before applying our randomization procedure, as described earlier. This may lead to a feature set associated with only very high activity sequences, as opposed to those associated with only marginally active sequences.

## Methods

### Dataset

When choosing sequences for our dataset, two things were considered absolutely necessary. First, any included sequence had to have a complete phosophothioate backbone, and no chimeric sequences were allowed. Second, the cellular level of antisense activity was assayed using either a protein product method or a direct mRNA method. The 3913 sequences used and their activities were obtained from previously published sources [20] as well as from the USPTO database [21]. The sequence/activity database, as well as the code used for the experiments, can be obtained from the authors at [22].

### Statistical calculations

This study uses a randomization procedure to determine which motifs are significantly associated with antisense activity. First, the dataset was separated into one group of 944 high activity sequences (activity in the range [0.0, 0.5]) and another group of 2924 low activity sequences (activity in the range (0.5, 1.0]). These two groups were analysed separately to determine sequences that associate significantly with either high or low antisense activity. First, the underlying base distribution of the data is obtained by counting the numbers of each type of base in all of the sequences in the dataset. The distribution of lengths and sequence activities are obtained similarly. These base, length, and activity distributions are then used to generate datasets of random sequence/activity pairs that have the same underlying distributions as the original dataset. Then, the number of each motif of interest in the effective sequences are counted and compared to the counts in the randomly generated effective sequences. If the number of occurrences of a motif in the original data exceeds that of the random data, it is recorded. These counts are also taken, compared, and recorded for the ineffective sequences in both datasets. This process is repeated a large number of times (for this study 10,000), and the proportion of times a motif occurs more often than random is calculated. Statistically speaking, this proportion is a confidence value that we can use to determine if a particular motif associates significantly with antisense activity. If the confidence value is greater than a threshold value (.975 for a two-tailed test, for example), and the counts came from the effective sequences, then we say that the motif is significantly associated with high antisense activity, or more succinctly it is a "good motif". We look at similar proportions from the counts using the ineffective sequences to determine the "bad motifs".

The limitations to this method are the same limitations faced by all randomization procedures, namely that the significance result applies only to this particular dataset. We must make the assumption that this dataset is representative of the true distribution of antisense sequences and activities in order to use the information garnered from this data to build a model for all antisense sequences, not merely those in our dataset.

Once the significant motifs are found, we wanted to determine if there was any association between the location of motifs within an antisense sequence and its activity. Since our motifs and our antisense sequences are comprised of different lengths, positional information was normalized to the same scale so that consistent comparisons could be made. To determine the positional distribution of a motif within the antisense dataset, the absolute position of the motif is divided by the maximum possible position that the motif could be located at. For example, to determine the positional distribution of the motif 'ACT' within the antisense sequence 'ATACTTGGTACTGTT', we note that, using 0 as our starting index, the motif of interest is located both at position 2 as well as position 9 within the antisense sequence. Since our motif has length 3, the maximum possible value for it is position 12, the largest index that allows the sequence to contain the entire motif. Thus, the locations for our motif of interest are each divided by 12, giving us a distribution of (0.167, 0.75). This can be repeated for other antisense sequences, giving us a position distribution for the motif over a group of antisense sequences. With this method, a value of 0 indicates the motif is located at the beginning of a sequence, while 1 indicates the motif is at the end of the sequence. Fractional values indicate an internal position for the motif of interest. Two positional distributions were calculated for each significant motif, one within the effective sequences and one within the ineffective sequences. These distributions were then used to compile 2-by-2 contingency tables with one axis given by sequence effectiveness and the second axis given by position within the sequence, either in the inner part or the outer part. Positions between 0.25 and 0.75 were considered to be in the inner portion, while positions outside of that range were considered to be in the outer portion. A chi-square test was performed on sufficiently large tables to determine any significant nonuniform association between motif position and sequence activity.

### Machine Learning and Prediction

Support vector machine calculations were made using the Python version of the LIBSVM package for Support Vector Machines [23]. Seven separate feature sets were constructed in order to test the efficacy of the significantly associated motifs as features for support vector regression (SVR). One feature set contained all possible motifs of length two to five, one contained the significant motifs, and one contained the subword-unique significant motifs derived from the significant motifs. The remaining four datasets were constructed by separating the significant and subword-unique significant sets according to association with effective or ineffective antisense activity. These seven feature sets were each used along with the empirical data to train an SVR algorithm to predict the activity of the entire dataset, as well as to train and predict under 10-fold cross-validation. The training vectors contained an entry for each of the motifs in the given feature set, which was incremented by 1 if that entry's motif was found in a search of the training sequence. Multiple occurrences of the same motif within a training sequence were counted. The data vector was then scaled to a maximum value of 1. These vectors were then associated with the activity value corresponding to the training sequence and used as the training data for the SVR algorithm. Correlation coefficient values and mean squared errors were recorded for each set of predictions, and then a *t*-test for correlation coefficients was used to see which feature sets, if any, gave significantly higher correlation than the others.

### Thermodynamic Calculations

Change in equilibrium Gibbs free energies (*δG*) were calculated by the nearest neighbour method [24] for change in Gibbs free energy

## Authors' contributions

KM wrote the code for the randomization procedure, ran the experiment, performed the statistical analysis, and drafted the manuscript. AP conceived the study and consulted on the drafting of the manuscript.
